# Draft Genome Sequence of *Pseudonocardia* *autotrophica* Strain DSM 43083, an Efficient Producer of Peroxidases for Lignin Modification

**DOI:** 10.1128/genomeA.01562-16

**Published:** 2017-02-02

**Authors:** Christian Grumaz, Dominik Rais, Philipp Kirstahler, Yevhen Vainshtein, Steffen Rupp, Susanne Zibek, Kai Sohn

**Affiliations:** a Fraunhofer Institute for Interfacial Engineering and Biotechnology, Stuttgart, Germany; b Institute of Interfacial Process Engineering and Plasma Technology, University of Stuttgart, Stuttgart, Germany

## Abstract

*Pseudonocardia autotrophica* strain DSM 43083 is a filamentous actinobacterium and was described to degrade or modify lignin. Here, we present its draft genome sequence, with a size of 5.8 Mb, to unravel the gene set coding for promising monooxygenases, dioxygenases, and DyP-type peroxidases associated with aromatic metabolism and lignin modification.

## GENOME ANNOUNCEMENT

The most abundant terrestrial biomass occurs as plant cell walls and consists of the sugar polymers cellulose and hemicellulose and the aromatic polymer lignin (1). Lignin is the largest potential renewable source for aromatic building blocks (2). Besides the prominent fungal lignin degraders, particularly the white-rot fungi, several bacteria strains are known to degrade or modify lignin as well. By investigating a wide range of organisms which are active against lignin and are able to metabolize lignin breakdown products, especially aromatic molecules, the general microbial strategies of lignin modification may be revealed (3). Understanding this lignin modification and metabolizing system will open new areas of applications and increase the efficient utilization of biomass in lignocellulosic biorefineries (4, 5).

Using a fluorescence assay, *Pseudonocardia autotrophica* was found to degrade lignin in the presence of hydrogen peroxide (6). Moreover, peroxidase activities were found in the culture supernatant, when *P. autotrophica* was cultivated in the presence of lignin (7). Here, we describe the draft genome sequence of the ligninolytic *P. autotrophica* strain DSM 43083. For this purpose, extracted DNA was prepared for Illumina HiSeq 2500 with the Nextera DNA kit using a standard protocol. Sequencing was performed in paired-end mode with 2 × 250 cycles, resulting in 3,077,959 read pairs and a genome coverage of 264×. Illumina reads were removed for contamination and adapter sequences with BBDuk from the BBMap package version 34.41 (http://sourceforge.net/projects/bbmap/). The genome was assembled using ABySS (version 1.5.2) generating 30 contigs, with an *N*_50_ of 346 kb and a largest contig size of roughly 1.2 Mb.

Assembled sequences have a total of 5.8 Mb, with a G+C content of 74.2%. Gene annotation was carried out using Prokka 1.11 (8) providing 5,390 potential open reading frames (ORFs). To unravel the genetic background of this ligninolytic machinery, we searched for characteristic enzyme domains of ligninolytic DyP-type peroxidases and genes associated with the metabolism of aromatic compounds.

The *in silico* search for oxidoreductases, which are related to the degradation of aromatic compounds and lignin, resulted in a set of genes with at least five relevant dioxygenases, three monoxygenases, and two DyP-type peroxidases. The predicted DyP-type peroxidases (EC 1.11.1.-) may be involved in the lignin degradation of bacteria and act on lignin (9). A putative dioxygenase is predicted to oxidize lignin-derived biphenyl molecules (EC 1.14.12.18), and other identified dioxygenases likely cleave aromatic rings in catechol, homogentisate, and protocatechuic acid (EC 1.13.11.-, EC 1.13.11.5, and EC 1.13.11.3/8, respectively). Furthermore, several putative monooxygenases are expected to hydroxylate salicylate, phenol, and *p*-hydroxybenzoate (EC 1.14.13.172, EC 1.14.13.7, and EC 1.14.13.2, respectively). The identified gene sequences can now be heterologously expressed and may be applied as tools for lignin degradation and modification of lignin-derived aromatic compounds.

### Accession number(s).

This whole-genome shotgun project has been deposited at DDBJ/EMBL/GenBank under SUBID SUB1911606 and BioProject PRJNA341362, BioSample SAMN05718038, and accession number MIFY00000000. The version described in this paper is version MIFY01000000.
